# West Indian Punctate Keratopathy and “Puerto Rican (PR) Spots”

**DOI:** 10.7759/cureus.48407

**Published:** 2023-11-06

**Authors:** Estefania Ramirez Marquez, Angel Aguayo, Sebastian Ruiz, Alejandra Santiago, Carmen Santos

**Affiliations:** 1 Ophthalmology, University of Puerto Rico School of Medicine, Medical Sciences Campus, San Juan, USA

**Keywords:** wipk, puerto rico, cornea, uveitis, west indian punctate keratopathy

## Abstract

This study describes the clinical characteristics of a cohort of Hispanic patients living in Puerto Rico who were found to have West Indian punctate keratopathy (WIPK). This case also introduces the term “PR (Puerto Rican) spots” to describe the dots observed in the eyes of individuals with WIPK who have a documented history of residing in Puerto Rico. The methods of the study consist of a retrospective chart review of patients presenting with WIPK. The patient data were entered retrospectively into a new database and analyzed. Eighteen patients who had WIPK were identified. The median age at presentation was 60.5 years (range: 49-72); 61.1% were female. At presentation, only one patient had both eyes affected. The median number of PR spots on examination was 1 (range: 1-4). All the patients had a history of ocular disease, most frequently glaucoma (55.5%), and had lived in Puerto Rico for more than 40 years. A total of 33.3% of the patients were retired or unemployed at the time of their presentation. While the origin of these dots remains unclear, ongoing efforts to document and characterize WIPK and PR spots will persist, with the aim of enhancing our understanding of this clinical entity.

## Introduction

West Indian (Caribbean) punctate keratopathy (WIPK) is a distinctive ocular finding that warrants comprehensive discussion due to rarity in the medical literature, with only a handful of reports, and notably, none originating from the Greater Antilles region [1-4]. This condition is defined by the presence of one or more asymptomatic white spots within the cornea [1-6]. These white spots are often visible to the naked eye. Histopathological investigations have suggested that WIPK spots may be composed of lipids [2]. These dots predominantly affect the subepithelial layer of the cornea and may extend toward the stromal layer [3,6]. These lesions may be clinically distinguished from other conditions involving the cornea by the fact that the overlying epithelium remains clear [6]. Additionally, the spots may manifest in one or both eyes of affected individuals, and the number and size of these white spots can vary among patients [4]. While the spots can potentially be found in any region of the cornea, they are most frequently reported in the lower half [2-4,6].

An important aspect of WIPK is that it does not cause visual disturbances or discomfort to patients [2,4]. This asymptomatic nature can make it challenging to diagnose, as patients often present to ophthalmologists with concerns related to other ocular conditions [3]. Consequently, it is essential for eye care professionals to be aware of WIPK's clinical characteristics to differentiate it from other eye conditions.

Despite its distinctive clinical features, WIPK is not the only condition that can lead to white spots on the cornea [4]. Therefore, it is important for ophthalmologists to consider a comprehensive differential diagnosis. Conditions such as Thygeson's superficial punctate keratitis and various punctate disorders of the corneal epithelium, including those caused by viral (e.g., adenovirus) or bacterial (e.g., Neisseria) infections, should be taken into account when evaluating patients with similar findings [2-4].

The factor that most distinguishes WIPK is its geographic association [4]. According to our research, there have been no reports in the medical literature characterizing a cohort of WIPK patients originating from the Greater Antilles, which includes Puerto Rico. Given this unique geographical context, we aim to describe the clinical characteristics (upon presentation) of a cohort of Hispanic WIPK patients living in Puerto Rico. This article also proposes the term “PR (Puerto Rican) spots” to denote the dots observed in the eyes of WIPK patients who have a documented history of having lived in Puerto Rico.

## Materials and methods

We identified patients from a photo log of clinic visits spanning from 2012 to 2023, totaling 20,802 patient encounters. During this period, the identified spots were documented and photographed. These records originated from individuals who sought care at the private uveitis and cornea outpatient clinic, which is an integral part of the Department of Ophthalmology at the University of Puerto Rico School of Medicine. These records were reviewed to identify patients diagnosed with WIPK.

Within this dataset, the diagnoses of WIPK were established through the discerning expertise of one of our authors, C.S., who bears the credential of a certified cornea specialist. It is worth noting that WIPK, as a unique ocular entity, sets itself apart from other corneal lesions through the preservation of normal corneal epithelium, the absence of visual disturbance, or any other symptomatic manifestations. During this study, the diagnosis of WIPK was based on evaluating a variety of factors. These factors included the presentation of characteristic clinical indicators, a review of the patient's medical history, a detailed exploration of the patient's social history, and a comprehensive slit-lamp examination. Furthermore, additional tests were conducted for some patients as most of them had other ocular diseases or complaints. The selection of these additional tests was determined by the unique clinical presentation of each patient, contributing to a comprehensive and precise diagnostic approach. These ancillary tests included a complete blood count, comprehensive metabolic panel, inflammatory markers analysis, infectious disease tests, chest X-rays, dilated fundus examinations, optical coherence tomography, and fluorescein angiography, among others. The results of these tests contributed to the diagnostic process, significantly enhancing the overall accuracy and effectiveness of this study.

The data obtained from the review of medical records that aligned with the diagnosis of WIPK was transcribed into a structured database. This data repository was designed to accommodate slit lamp exam pictures as well as clinical and demographic information for subsequent in-depth analysis. The demographic attributes of these patients encompassed a multifaceted range of parameters, including age, gender, race, their prevailing occupational or employment status, ethnicity, and the cumulative duration of their residence in Puerto Rico, USA. Clinical parameters documented included the laterality of spots and the presence of ocular disease upon presentation. 

The dataset was organized and systematically tabulated, adhering to a structured approach that would enable a comprehensive descriptive statistical analysis. To ensure optimal analytical processes, the Microsoft Excel software program was specifically selected as the tool of choice for this study. The utilization of this software not only streamlined the data management process but also allowed for the application of various statistical functions and analytical methodologies, aiding in the in-depth exploration and interpretation of the collected data. The protocol for this study underwent a thorough evaluation and received the endorsement of the Institutional Review Board at the University of Puerto Rico Medical Sciences Campus.

In alignment with the ethical principles governing medical research, the Institutional Review Board of the University of Puerto Rico Medical Sciences Campus conducted a rigorous review of the research protocol. Following a thorough examination, the protocol was granted its unequivocal approval, underscoring the meticulous adherence to ethical standards and patient confidentiality that guided this comprehensive retrospective study.

## Results

**Figure 1:**
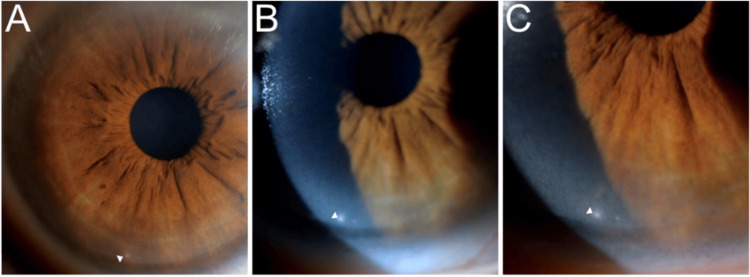
The right eye anterior segment photo with lateral indirect (A) and direct (B and C) slit-lamp illumination reveals inferior subepithelial white opacity (white arrowhead) measuring 0.1 mm vertically by 0.1 mm horizontally.

**Figure 2:**
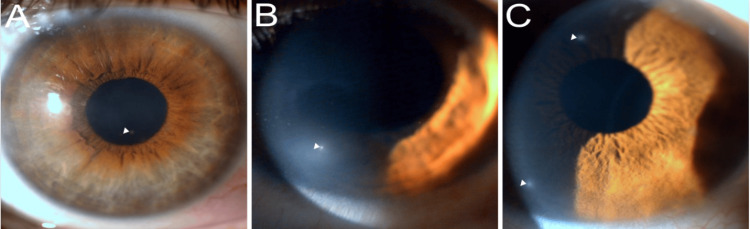
(A) The left eye anterior segment photo with lateral indirect slit-lamp illumination shows a paracentral white opacity (white arrowhead). (B) The right eye anterior segment photo with direct slit-lamp illumination demonstrates a paracentral white opacity (white arrowhead). (C) The right eye anterior segment photo with direct slit-lamp illumination demonstrates two paracentral white opacities (white arrowhead).

A cohort comprising a total of 18 patients with WIPK was identified during an extensive period of time (11 years). The patients’ demographic and clinical characteristics have been summarized (Table 1). The patient’s age at presentation ranged from 49 to 72 years; the median age at presentation was 60.5 years. Among those 18 patients, 11 were female (61.1%). All the individuals included in this cohort identified themselves as Hispanic. The total number of eyes with WIPK was 19 (52,7%). Patients with a single affected eye accounted for 94.5% of the group; only one patient (5.5%) had bilateral WIPK. In terms of the number of characteristic spots, the patients had from 1 to 4 spots; the median number at presentation was 1 spot (Figures 1, 2). 

**Table 1:** Demographic and clinical characteristics of patients with West Indian punctate keratopathy and PR spots. PR: Puerto Rican

Characteristic	Value
Median age (in years)	60.5
Gender (%)	
Male	38.8
Female	61.1
Race (%)	
Hispanic	100
Other	0
Years living in Puerto Rico (%)	
40 or fewer	0
More than 40	100
Occupational status (%)	
Unemployed or retired	33.3
Clerical worker	27.9
Teacher	22.2
Healthcare provider	11.1
Sanitation worker	5.5
Laterality of PR spots (%)	
Unilateral PR spots	94.5
Bilateral PR spots	5.5
Ocular disease at presentation (%)	
Glaucoma	55.5
Uveitis	44.4
Herpes simplex keratitis	16.6
Chronic conjunctivitis	5.5
Retinal detachment	5.5
Corneal ulcer	5.5
No ocular disease	0

All the patients included had a history of ocular disease. The ocular diseases of the patients included glaucoma (55.5%), uveitis (44.4%), herpes simplex keratitis (16.6%), chronic conjunctivitis (5.5%), retinal detachment (5.5%), and corneal ulcer (5.5%). Details of each patient’s social history, including how many years he or she had lived in Puerto Rico and his or her current occupation and/or occupational status, were also obtained. All the patients (100%) had lived for more than 40 years in Puerto Rico. The occupational status of the patients was being employed or being unemployed or retired (33.3%); those who were employed were clerical workers (27.9%), teachers (22.2%), healthcare providers (11.1%), and sanitation workers (5.5%). 

## Discussion

This is the first report of a group of patients in the Greater Antilles (including Puerto Rico, USA) with WIPK. The examinations of the 16 patients included in the study showed them to have round, white spots located at the level of the corneal subepithelium; such spots are characteristic of WIPK (Figure 1 and Figure 2). It is interesting to note that, in Florida, there have been similar, though larger, spots identified in cats and dogs which also do not affect visual acuity; these spots are called “Florida spots” [5,7,8]. Similarly, we introduce the term “PR spots,” derived from the phrase “Puerto Rican spots,” to specifically denote the dots observed in the eyes of WIPK patients who have a documented history of having lived in Puerto Rico. This report also aims to further understand and characterize WIPK. 

White spots in the cornea can result from a myriad of conditions other than WIPK [3,4,9]. Adenovirus, for instance, may lead to epidemic keratoconjunctivitis, a condition that presents with similar yet less dense subepithelial opacities [4,10]. Conditions such as Epstein-Barr virus keratitis and nummular keratitis can also cause round corneal lesions, but these are located at different levels within the corneal stroma. Examination of Thygeson’s superficial punctate keratopathy (TSPK) also reveals dots in the cornea [4,9,11,12]. However, TSPK primarily affects the corneal epithelium and tends to manifest with a range of symptoms, including photophobia, tearing, burning, and a foreign body sensation [13-15]. It is also important to consider non-infectious differential diagnoses such as scars in patients with a history of superficial corneal foreign bodies or ocular trauma. These can often be differentiated based on their epithelial involvement.

The PR spots in patients with WIPK are unique because they are asymptomatic and, notably, do not lead to any loss of visual acuity. These spots are situated within the corneal subepithelium, displaying a distinct white and opaque appearance. Interestingly, they are surrounded by corneal tissue that maintains its usual integrity, except in cases where a patient may have another corneal disease at the time of evaluation. Moreover, WIPK lesions exhibit long-term stability, setting them apart from other punctate keratopathies rooted in infectious causes that tend to evolve over time. It is also worth noting that, unlike other corneal diseases, medications have not been shown to induce any discernible changes in the appearance of the spots seen in WIPK, further underscoring their unique nature and clinical characteristics.

Studies have suggested that WIPK is usually found in patients who are adults rather than in those who are children, though there is no predilection for sex [2,4]. In our group, there were more females than males affected, and the median age at presentation was 60.5 years. A plausible explanation for the higher association of WIPK with older patients is that older individuals tend to have a higher likelihood of experiencing ocular diseases and may seek ophthalmic evaluation more frequently than children do [2-4,6]. Another possibility is that extended exposure to environmental risk factors over time among older individuals in Puerto Rico may contribute to the increased prevalence of WIPK in adults compared to children. Remarkably, we recently reported the youngest patient we have encountered with WIPK, which was the case of a 9-year-old girl with uveitis; however, she was seen at another one of our clinics that is not included in this study [1]. 

 Most of the patients in this group presented unilateral involvement. This aligns with findings from previous articles that have also emphasized a prevailing unilateral involvement trend in cases of WIPK [2-4,9]. The number of spots found on a given patient’s corneas varied from one to four, with most patients presenting a single spot. McLendon et al. (1993) also reported that the majority of their patients had a single dot [3]. 

All the patients included in this study had a history of ocular disease, with glaucoma and uveitis being the most prevalent conditions. However, it is important to note that PR spots have not yet been associated with any disease [4,10]. The asymptomatic nature of the condition may also lead to false associations with other diseases as asymptomatic patients do not always actively seek ophthalmic evaluation [2]. It would be interesting to explore the prevalence of the spots in patients without ocular disease or patients from a general ophthalmology clinic in Puerto Rico.

Out of a total of 20,802 cases reviewed, only 18 patients were diagnosed with WIPK. These patients were diagnosed at an outpatient uveitis and cornea clinic located on the island of Puerto Rico, which is part of the Greater Antilles, a Caribbean archipelago. Notably, the existing literature presents only three other cohorts involving patients with WIPK, including patients from Grenada, Colombia, and the British West Indies [3,4,9]. Among these, the study conducted in the general ophthalmology clinic in Grenada stands out as the largest, encompassing a total of 128 patients out of 2,048 cases with WIPK [3]. Interestingly, our study examined a substantial population of 20,802 individuals, yet we identified only 18 patients with WIPK. This notable difference in prevalence between Grenada and Puerto Rico prompts intriguing questions. It is plausible that an undisclosed risk factor might exert a more significant influence in Grenada compared to Puerto Rico, or it could be a coincidental variation worth further exploration.

The etiology of WIPK spots is uncertain, and there are various theories in the literature. The most prevalent include corneal microtrauma, factors associated with agricultural elements, and prolonged exposure to dust from the Sahara Desert carried by the wind across the Atlantic Ocean [2-4,8,10]. There are currently no reported data to support or disprove any of these theories. To test the previously stated theories, we gathered the following data from the study participants: the length of time that they had lived in Puerto Rico and their current occupational status and occupation type. All the patients had lived in Puerto Rico for more than 40 years. The patients’ leading occupational status was being unemployed or retired, and none of them reported having engaged in any activities related to agriculture. The nature of the dots under discussion remains uncertain; however, efforts to document PR spots will continue until the condition can be better characterized. 

While this study provides valuable insights into WIPK, it may be limited due to its retrospective nature, potentially introducing ascertainment and referral biases. Given that this investigation was carried out at a subspecialized clinic, its results may not be generalized to the broader population in Puerto Rico. Further research is warranted to conduct a comprehensive analysis of this condition.

## Conclusions

In conclusion, this study marks a significant milestone as the first report of WIPK in the Greater Antilles, specifically in Puerto Rico, USA. The clinical examinations of the 18 patients included in this study unequivocally confirmed the presence of characteristic round, white spots within the corneal subepithelium, which are a hallmark feature of WIPK. In response, we propose the term "PR spots," derived from "Puerto Rican spots," to specifically describe these dots in WIPK patients with a documented history of residence in Puerto Rico. Our study reinforces previous findings that WIPK predominantly affects adults. This observation may be attributed to the greater likelihood of ocular diseases in older individuals, prompting more frequent ophthalmic evaluations. While this cohort supports the predominant unilateral involvement and presentation with a single spot. Notably, all patients in this study had a history of ocular diseases, with glaucoma and uveitis being the most common coexisting conditions; however, PR spots themselves have not been linked to any specific disease. The etiology of WIPK spots remains elusive, with hypotheses ranging from corneal microtrauma to environmental factors like agricultural elements and Sahara Desert dust exposure, though none have been conclusively proven. Our data suggest that the patients had resided in Puerto Rico for an extended period, with most being retired or unemployed and not engaged in agricultural activities. As we continue to explore and document PR spots, it is clear that further research is essential to unravel the mysteries surrounding this unique ocular condition and better characterize its origins and implications.
